# Repurposing Glutathione Transferases: Directed Evolution Combined with Chemical Modification for the Creation of a Semisynthetic Enzyme with High Hydroperoxidase Activity

**DOI:** 10.3390/antiox13010041

**Published:** 2023-12-25

**Authors:** Irene Axarli, Farid Ataya, Nikolaos E. Labrou

**Affiliations:** 1 Laboratory of Enzyme Technology, School of Applied Biology and Biotechnology, Agricultural University of Athens, 75 Iera Odos Street, GR-11855 Athens, Greece; eaxarli@aua.gr; 2 Department of Biochemistry, College of Science, King Saud University, P.O. Box 2455, Riyadh 11451, Saudi Arabia; fataya@ksu.edu.sa

**Keywords:** chemical modification, directed evolution, enzyme engineering, glutathione transferase, glutathione peroxidase

## Abstract

Glutathione peroxidases (GPXs) are antioxidant selenoenzymes, which catalyze the reduction of hydroperoxides via glutathione (GSH), providing protection to cells against oxidative stress metabolites. The present study aims to create an efficient semisynthetic GPX based on the scaffold of tau class glutathione transferase (GSTU). A library of GSTs was constructed via DNA shuffling, using three homologue GSTUs from *Glycine max* as parent sequences. The DNA library of the shuffled genes was expressed in *E. coli* and the catalytic activity of the shuffled enzymes was screened using cumene hydroperoxide (CuOOH) as substrate. A chimeric enzyme variant (named Sh14) with 4-fold enhanced GPX activity, compared to the wild-type enzyme, was identified and selected for further study. Selenocysteine (Sec) was substituted for the active-site Ser13 residue of the Sh14 variant via chemical modification. The GPX activity (k_cat_) and the specificity constant (k_cat_/Κ_m_) of the evolved seleno-Sh14 enzyme (SeSh14) was increased 177- and 2746-fold, respectively, compared to that of the wild-type enzyme for CuOOH. Furthermore, SeSh14 effectively catalyzed the reduction of hydrogen peroxide, an activity that is completely undetectable in all GSTs. Such an engineered GPX-like biocatalyst based on the GSTU scaffold might serve as a catalytic bioscavenger for the detoxification of hazardous hydroperoxides. Furthermore, our results shed light on the evolution of GPXs and their structural and functional link with GSTs.

## 1. Introduction

Glutathione peroxidases (GPXs, EC.1.11.1.9) are selenoenzymes that catalyze the reduction of hydroperoxides via glutathione (GSH), which serves as a reducing substrate [1,2,3]. GPXs are essential components of the cell’s antioxidant defense system since they offer protection from oxidative stress. Their activity depends on the rare amino acid residue selenocysteine (Sec) in the active site, which is essential for the antioxidant activity [4,5,6]. During the catalytic cycle, selenenic acid is formed, which is converted to selenenyl sulfide by GSH. The reaction between selenenyl sulfide with a second equivalent of GSH leads to the formation of selenol. Since Sec is encoded by the stop codon UGA, the production of GPXs using recombinant DNA technology poses a considerable challenge. However, chemical modification or genetic engineering strategies using auxotrophic expression systems can be used for the incorporation of Sec into natural enzymes [7,8,9,10].

The cytosolic glutathione transferases (GSTs, EC. 2.5.1.18) catalyze the conjugation of GSH with a broad range of electrophilic compounds [11,12,13]. They comprise a highly versatile superfamily that is divided into several different classes. The plant GST subfamily has been well characterized and is divided into eight groups: Phi, tau, zeta, theta, lambda, dehydroascorbate reductase, and tetrachlorohydroquinone dehalogenase [12]. The substantial scientific interest in the tau class GSTs (GSTUs) is primarily attributed to their participation in various biotic and abiotic stress response mechanisms as well as regulatory activities [12,14,15,16]. For example, GSTUs play key roles in herbicide metabolism and selectivity as well as the protection against oxidative stress metabolites [16].

GSTs display a wide substrate specificity, particularly towards hydrophobic molecules such as organic halides, epoxides, arene oxides, α- and β-unsaturated carbonyls, organic nitrate esters, and organic thiocyanates. These enzymes not only facilitate the conjugation of GSH to electrophilic compounds, but they also possess additional functions. For instance, they play a role in the biosynthesis of prostaglandins and in glutathione-dependent isomerization reactions [16,17,18]. Members of the GST family display high hydroperoxidase activity and are able to catalyze the reduction of organic hydroperoxides to their respective less toxic alcohols [12,16]. However, GSTs do not display any activity with hydrogen peroxide, the natural substrate of GPXs [1,3,12,14,16].

GSTs are dimeric proteins able to form homodimers or heterodimers. Each monomer is composed of two domains, namely the small α/β domain and the large β-helical domain [13,14,15,16,17,18]. A GSH binding site (G-site) is located on top of the α/β domain, while a hydrophobic pocket (H-site) that binds the electrophilic substrate overlaps the two domains [12,13]. The G-site is specific for GSH. On the other hand, the H-site displays remarkable structural diversity, plasticity, and flexibility. This unique characteristic enables GSTs to exhibit catalytic promiscuity and they are able to bind a broad spectrum of substrates with varying structures. Furthermore, apart from their role in detoxification, GSTs also exhibit non-catalytic ligand-binding function [12,13,14]. They have the capability to bind hydrophobic xenobiotic or endogenous molecules at a specific site known as the L-site [15]. This non-catalytic function enables the sequestration, storage, or transportation of these compounds to specific intracellular targets, including protein receptors [16].

Both GSTs and GPXs belong to the thioredoxin superfamily, which also includes thioredoxin, glutaredoxin, and disulfide-bond formation facilitator [13,19]. Their classification is based on the presence of a common GSH-binding domain, which adopts the thioredoxin fold [13,19]. The active-site residues of GSTs and GPXs are located in similar positions at the N-terminus domains. The active-site residue in GPX is Sec; however, in GSTs, it is Tyr, Ser, or Cys. Taking into account these structural similarities, GSTs may provide ideal protein scaffolds for engineering GPX activity by introducing a Sec residue into the G-site. Previous investigations have shown some success such as, for example, the conversion of GSTT2-2 or GSTZ1-1 via the chemical modification of the active-site Ser to Sec [7,8,9,10].

In the past two decades, a large number of structural and functional studies on GSTs allowed for the accumulation of in-depth scientific knowledge, paving the way for the design of tailor-made GST variants with desired properties [20,21,22,23,24,25,26,27,28,29,30,31,32]. The GST scaffold appears to be a very flexible and amenable platform for designing novel catalytic activities [12]. The features of the GST scaffold that make it a suitable tool for engineering studies can be summarized as follows: (a) GSTs display highly diverse functions and specificities, being promiscuous enzymes and able to catalyze the transformation of a wide spectrum of substrates [24,29,32]; (b) they have a conserved modular architecture with well-organized domains with distinct binding pockets (e.g., G-site, H-site, and L-site) [12,14,15]; (c) recombinant GSTs can be produced in a high yield in *Escherichia coli* and purified in a single chromatographic step using affinity methods [20,21,22,23,24,25,26,27,28,29,30]; (d) the structural biology of GSTs is well studied with several high-resolution structures available (ligand-free or ligand-bound [12,13,17,33]; and (e) GSTs display sufficient operational stability, with a melting temperature (T_m_) between 50 and 65 °C [34,35].

In the present work, an efficient approach, relying on directed enzyme evolution and chemical modification, was applied for the creation of an improved GST variant with high GPX activity. A library of GSTUs was created via DNA shuffling. The library was activity screened, allowing for the selection of an enzyme variant with about 4-times higher hydroperoxidase activity towards CuOOH. This variant was further modified using chemical modification, leading to a novel selenium-containing enzyme with dramatically increased GPX activity towards CuOOH as well as hydrogen peroxide. The results of the present study provide further insightful information to support the notion that GST and GPX diverged from a common thioredoxin-like progenitor to fulfill different roles during their evolutionary histories [12,14,18]. Furthermore, our work establishes a methodology and resources for the design and production of novel GST variants able to cope with oxidative stress and its harmful metabolites.

## 2. Materials and Methods

### 2.1. Materials

Reduced GSH, 1-chloro-2,4-dinitrobenzene (CDNB), ampicillin and kanamycin were obtained from Sigma-Aldrich, (Sigma-Aldrich Co., St. Louis, MO, USA). Glutathione disulfide (GSSG) was obtained from Roche (Roche Diagnostics, Manheim, Germany). Glutathione reductase (type III baker’s yeast), reduced nicotinamide adenine dinucleotide phosphate (NADPH), cumene hydroperoxide (CuOOH), tert-butyl hydroperoxide (t-BuOOH), hydrogen peroxide (H_2_O_2_), and phenylmethanesulfonyl fluoride (PMSF) were obtained from Sigma-Aldrich, (Sigma-Aldrich Co., St. Louis, MO, USA). All the other reagents were of analytic grade.

### 2.2. Methods

#### 2.2.1. DNA Shuffling and Construction of GmGSTUs Library

Three isoenzymes from *Glycine max* (*Gm*GSTU2-2, *Gm*GSTU4-4, and *Gm*GSTU10-10) [15,34,35] were used as parent sequences for directed evolution using the DNA shuffling method [14]. Shuffled library was constructed as described previously [14]. For activity screening, transformants were grown at 37 °C in LB medium (10 mL), and the hydroperoxidase activity was measured using CuOOH as a substrate as described previously [15].

#### 2.2.2. Expression and Purification of Recombinant Enzymes

The wild-type and shuffled *Gm*GSTUs were expressed in *E. coli* M15[pREP4] cells at 37 °C in LB medium containing ampicillin (100 μg/mL) and kanamycin (50 μg/mL) as described in [14,15]. Enzyme purification was carried out using affinity chromatography as described previously [14,15]. Protein purity was judged by SDS-PAGE.

#### 2.2.3. Assay of Enzyme Activity and Kinetic Analysis

Enzyme assays using 1-chloro-2,4-dinitrobenzene (CDNB) were performed according to published methods [14,15]. Observed reaction velocities were corrected for spontaneous reaction rates when necessary. Protein concentration was determined by the Bradford assay using BSA as standard. The GPX activity was assayed using the coupled assay described in [15]. The GST activity towards CuOOH was determined in sodium phosphate buffer (50 mM, pH 7.0) containing 1 mM EDTA, 1.0 mM GSH, and 1.5 mM CuOOH. Steady-state kinetic measurements were performed as previously described [16]. The Michaelis–Menten equation was fitted to the steady-state data via nonlinear regression analysis using the GraphPad Prism version 7 (GraphPad Software Inc., Boston, MA, USA) or GraFit version 4 (Erithacus Software Ltd., Sussex, UK).

#### 2.2.4. Site-Directed Mutagenesis

Site-directed mutagenesis was carried out as described [36]. The mutant Ser12Ala was created as described in [36]. The pairs of oligonucleotide primers used in the PCR reactions for the creation of Cys114Trp, Cys114Phe, Cys114Leu, Cys114Ala mutant enzymes of Sh14 are listed in Supplementary Table S1.

#### 2.2.5. Chemical Modification for the Creation of Selenium-Containing Enzymes

The wild-type *Gm*GSTU4-4, the Ser13Ala mutant, and the Sh14 shuffled enzyme (0.1 mg, 1 mL) previously dialyzed in potassium phosphate buffer (20 mM, pH 7.0) were mixed with PMSF (2–5 mg, dissolved in acetonitrile) and allowed to react at 25 °C for 3 h. The resulting solution was mixed with an equal volume (1 mL) of 0.58 M sodium hydrogen selenide solution prepared as described previously [37] and was then incubated at 25 °C for 20 h under nitrogen atmosphere. The selenium-containing enzymes were purified from unreacted chemicals using Sephadex G-25 chromatography. 

## 3. Results and Discussion

### 3.1. Directed Evolution of Three Homologous GmGSTUs for the Isolation of an Enzyme Variant with High Hydroperoxidase Activity

**Figure 1:**
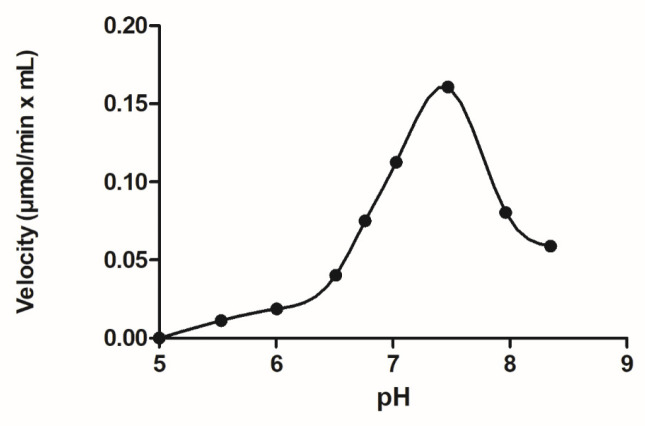
Dependence of the initial velocity of *Gm*GSTU4-4 on pH. Enzyme activity measurements were performed using GSH and CuOOH as substrates using the standard assay [15].

An interesting catalytic feature of GSTUs is their hydroperoxidase activity, being able to catalyze the reduction of the model substrate CuOOH to the respective alcohol with the concomitant oxidation of GSH to glutathione disulfide (GSSG) [16,18,35,36]. The tau class isoenzymes *Gm*GSTU2-2, *Gm*GSTU4-4, and *Gm*GSTU10-10 from *Glycine max* display high hydroperoxidase activity [15,34,35] with CuOOH as a substrate; however, the enzymes are not catalytically active with H_2_O_2_, the natural substrate of GPXs. The mechanism of GSTs functioning as hydroperoxidases is not identical to that displayed by GPX. For example (Figure 1), the optimum pH of *Gm*GSTU4-4 using CuOOH as a substrate is approximately 7.5, very close to the optimum pH of GSTs acting as transferases with CDNB and GSH as substrates [38]. On the other hand, GPXs display optimal pH in the alkaline range (pH 8.8) [39], where *Gm*GSTU4-4 shows very low activity.

**Figure 2:**
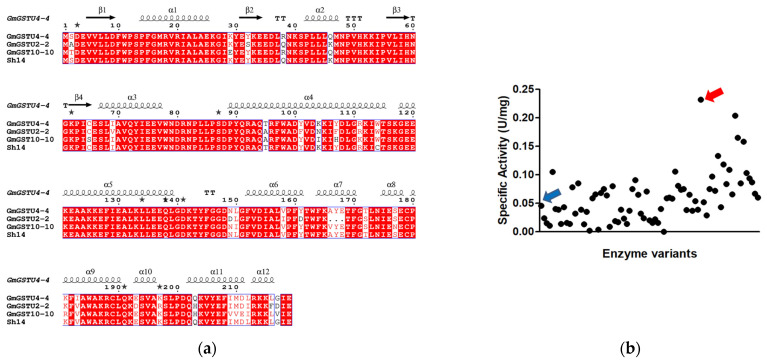
(**a**) Alignments of the *Gm*GSTU2-2, *Gm*GSTU4-4, and *Gm*GSTU10-10 parent sequences with the sequence of the Sh14 engineered variant. The figure was produced using ESPript [40]. *Gm*GSTU4-4 numbering is shown above the alignment. The secondary structure of *Gm*GSTU4-4 (PDB code 2VO4) is shown at the top. Beta turns are marked with TT. Conserved areas are shown shaded. A column is framed if more than 70% of its residues are similar according to the physicochemical properties. Stars on the top of sequences represent residues with alternate positions. NCBI accession numbers for sequences are in parentheses: *Gm*GSTU4-4 (AAC18566), *Gm*GSTU2-2 (CAA71784), and *Gm*GSTU10-10 (AAG34800.1); (**b**) activity screening of the *Gm*GSTUs library. The wild-type enzyme (blue arrow) as well as members of the library were assayed using CuOOH as substrate. The enzyme variant (Sh14) with the highest specific activity (red arrow) was selected for further study.

Recombination of the cDNA from three homologous GSTUs (*Gm*GSTU2-2, *Gm*GSTU4-4, and *Gm*GSTU10-10) via DNA shuffling [14] produced a library of GST variants. The amino acid sequences of the three parent GSTs differ by no more than 11.1% (Figure 2a); however, their catalytic activity towards a range of diverse xenobiotic substrates differs by more than 500-fold [14,15,34,35]. Therefore, these homologue *Gm*GSTUs offer an attractive group of proteins for studying enzyme activity within a very narrow range of primary structure diversity.

Following in vitro recombination, activity screening was used to assay about 100 different colonies for hydroperoxidase activity using CuOOH as a substrate. The results of the screening experiment revealed one enzyme variant that exhibited >4-times higher activity in crude lysate (Figure 2b), compared to the wild-type enzymes, and was selected for further characterization. Sequence analysis of the enzyme variant revealed that it is identical to that found (herein designated Sh14) by Axarli et al. [14] using CDNB or fluorodifen as a substrate. The Sh14 variant (Figure 2a) is a derivative of the *Gm*GSTU4-4 enzyme and possesses three substituted segments from *Gm*GSTU2-2 and *Gm*GSTU10-10, which led to three point mutations (Arg38Gln, Gln46Lys, Ile183Val) and a random point mutation (Trp114Cys), presumably generated by polymerase (Figure 2a).

**Figure 3:**
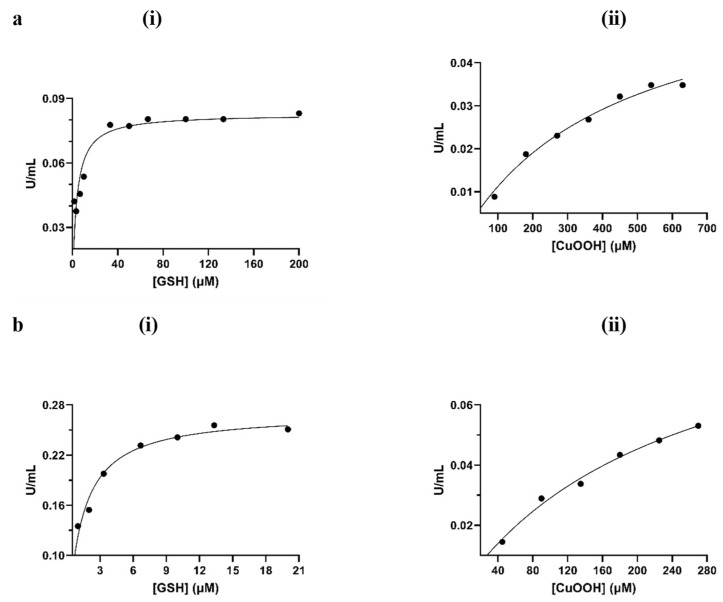
Kinetic analysis of *Gm*GSTU4-4 and Sh14 towards GSH and CuOOH. (**a**) Kinetic analysis of *Gm*GSTU4-4 enzyme using the GSH (1.66–200.0 μΜ) as a variable substrate (i) and CuOOH at a fixed concentration (1.5 mM). Kinetic analysis of *Gm*GSTU4-4 using the CuOOH (90.0–630.0 μΜ) as a variable substrate (ii) and GSH at a fixed concentration (1.0 mM); (**b**) kinetic analysis of Sh14 enzyme using the GSH (1.0–20.0 μΜ) as a variable substrate (i) and CuOOH at a fixed concentration (1.5 mM). Kinetic analysis of Sh14 using the CuOOH (45.0–225.0 μΜ) as a variable substrate (ii) and GSH at a fixed concentration (1.0 mM). Steady-state data were analyzed via nonlinear regression analysis using the GraphPad Prism 7.00 (GraphPad Software).

The Sh14 variant, as well as the wild-type *Gm*GSTU4-4 enzyme, was purified on a GSH-Sepharose column and subjected to steady-state kinetic analysis using CuOOH and GSH as substrates. The results are shown in Figure 3 and the measured kinetic parameters are listed in Table 1. The outcome of the kinetics analysis showed that both enzymes obey Michaelis–Mentel kinetics towards CuOOH or GSH. Axarli et al. [14] reported that the Sh14 enzyme, when assayed using fluorodifen or CDNB as variable substrates, exhibited sigmoidal kinetics. This allosteric behavior of Sh14 was interpreted as a consequence of structural adjustments, which strengthened the interaction of the two salt bridges at the dimer interface, between Glu66 and Lys104. These electrostatic interactions induced an allosteric effect by facilitating the interaction and structural communication between the two adjusted H-sites [15].

**Figure 4:**
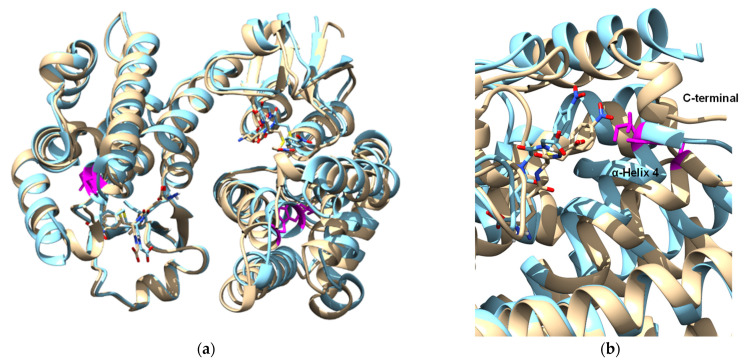
(**a**) Superposition of the structure of *Gm*GSTU4-4 (brown) with Sh14 (blue) variant. The side chains of Cys114 and Trp114 are shown in a stick representation and colored magenta. The inhibitor 4-nitrobenzyl-GSH bound to the wild-type (brown) and sh14 variant (blue) is shown in a stick representation; (**b**) conformational changes at the C-terminal and in α-helix 4. The side chains of Cys114 and Trp114 are shown in a stick representation and colored magenta. The inhibitor 4-nitrobenzyl-GSH bound to the wild-type (brown) and Sh14 variant (blue) is shown in a stick representation. The figures were created using the UCSF chimera software (https://onlinelibrary.wiley.com/doi/10.1002/jcc.20084) [41].

**Figure 5:**
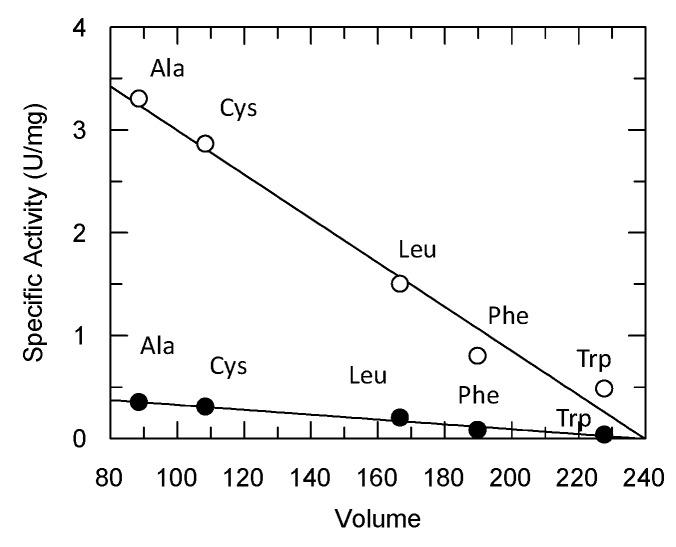
The dependence of amino acid volume (Å^3^) at position 114 on the specific activity (U/mg) of the Sh14 variant (114Cys) and of its mutant enzymes (114Trp, 114Phe, 114Leu, 114Ala). Assays were carried out using fluorodifen (●) or CuOOH (○) as substrates.

The structure of Sh14 has been determined at 1.75 Å resolution in a complex with S-(p-nitrobenzyl)-glutathione [14]. The Trp114Cys point mutation has been identified as being responsible for the altered kinetic properties of the enzyme (Figure 4). It was hypothesized that the replacement of the bulky Trp residue at the H-site with the smaller Cys led to a conformational change in α-helix 4 that affects the size and the volume of the H-site. To further evaluate this hypothesis, a group of site-directed mutants of Sh14 at position 114 were created and their specific activity was analyzed (Figure 5). Four different amino acid residues (Trp, Phe, Leu, Ala) with different sizes were selected for replacing Cys114. Interestingly, the results showed the existence of a linear dependence (R^2^ 0.98) between the specific activity towards two different substrates (the pesticide fluorodifen and CuOOH) versus the volume (Å^3^) of the amino acid residue at position 114. In particular, the mutants with a bulky side chain at position 114 (Trp, Phe) exhibit lower specific activities, compared to the mutant with a smaller side chain, such as Ala. Leu shows an intermediate specific activity in agreement with its volume. Another observation that can be drawn from the data in Figure 5 is that the specific activity of the mutants is not affected by the side-chain polarity of the residues at position 114. These observations point to the conclusion that the volume/size of the amino acid residue at position 114 is the main determinant for the enhanced activity of the Sh14 variant.

### 3.2. Chemical Modification for the Creation of Selenium-Containing Enzymes

**Figure 6:**
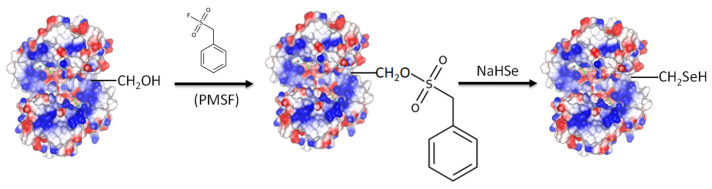
Schematic representation of the reactions for the generation of selenoenzymes. The active-site Ser13 is modified by phenylmethylsulfonyl fluoride (PMSF) and the hydroxyl group of Ser is converted to a sulfonyl ester. Next, the sulfonyl group is replaced by sodium hydrogen selenide and the selenoenzyme is formed.

As demonstrated by previous investigations [7,8], selenocysteine (Sec) can be effectively incorporated into an enzyme’s active site using the chemical modification of Ser residues. Preparing tailored enzymes using conventional recombinant DNA technology is a difficult task since Sec is encoded by the stop codon UGA [9,10]. Here, we report the conversion of *Gm*GSTU4-4 and Sh14 to selenoenzymes by means of chemical modification of Ser13 (Figure 6). The Ser13 in the active site of the *Gm*GSTU4-4 and Sh14 was initially modified by phenylmethylsulfonyl fluoride (PMSF) and the resulting modified enzymes were mixed with sodium hydrogen selenide to yield seleno-*Gm*GSTU4-4 (Se*Gm*GSTU4-4) and seleno-Sh14 (SeSh14). As a control reaction, the active-site mutant Ser13Ala [36] of *Gm*GSTU4-4 was treated (PMSF and sodium hydrogen selenide) exactly as the wild-type enzyme for assessing the specificity of the chemical modification reaction.

All selenoenzymes were purified, and their activity was measured using CuOOH and hydrogen peroxide as substrates. Se*Gm*GSTU4-4 and SeSh14 showed zero activity using CDNB/GSH as substrates, suggesting that both enzymes have lost their native glutathione transferase activity. Previous investigation has established the crucial role of Ser13 in the catalytic mechanism of *Gm*GSTU4-4 using site-directed mutagenesis [36]. In particular, it has been concluded that Ser13 directly contributes to the nucleophilic substitution reaction and to the correct positioning of GSH and CDNB in the ternary catalytic complex [36]. It is well established that the catalytic activities of GSTUs are based on the ability of these enzymes to decrease the pKa of the sulfydryl group of reduced GSH from 9.0 in an aqueous solution to approximately 6.5 when GSH is bound in the active site. Several crystal structures have demonstrated that an active-site Ser residue, Ser13 in the case of *Gm*GSTU4-4, forms a hydrogen bond with the sulfur atom of GSH. Ser13 is positioned in a manner that enhances the stabilization of the thiolate anion of GSH, leading to its higher nucleophilicity [14,15,33,34,35,36].

**Figure 7:**
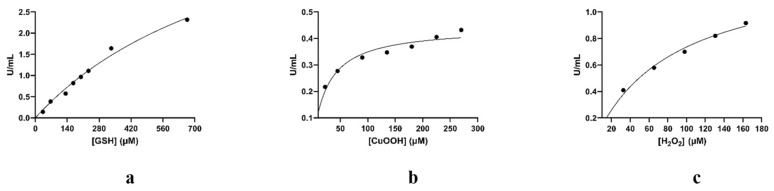
Kinetic analysis of SeSh14 towards GSH, CuOOH, and H_2_O_2_. (**a**) Kinetic analysis of SeSh14 using GSH (33.0–666.0 μΜ) as a variable substrate and CuOOH at a fixed concentration (1.5 mM). The concentration of enzyme used was 8.5 μg/mL. (**b**) Kinetic analysis of SeSh14 using CuOOH (22.5–270.0 μΜ) as a variable substrate and GSH at a fixed concentration (0.5 mM). The concentration of enzyme used was 1.3 μg/mL. (**c**) Kinetic analysis of SeSh14 using H_2_O_2_ (32.6–163.2 μΜ) as a variable substrate and GSH at a fixed concentration (0.5 mM). The concentration of enzyme used was 2.8 μg/mL. Steady-state data were analyzed via nonlinear regression analysis using the GraphPad Prism 7.00 (GraphPad Software).

Both enzymes displayed high hydroperoxidase activity towards CuOOH and were able to reduce H_2_O_2_ similar to that catalyzed by the native GPX (Table 2). On the other hand, the seleno-Ser13Ala (SeSer13Ala) did not display significant hydroperoxidase activity (Table 2). Interestingly, the SeSer13Ala mutant enzyme exhibited a dramatic reduction in activity towards all the tested substrates (CDNB, CuOOH, and H_2_O_2_), underlining the crucial role of Ser13. Kinetics analysis of the SeSh14 showed that it obeys Michaelis–Mentel kinetics using GSH, CuOOH, and H_2_O_2_ as substrates (Figure 7a–c; Table 1). Between the two enzymes, the magnitude of the hydroperoxidase activity of the SeSh14 appears to be superior compared to the Se*Gm*GSTU4-4. These features differentiate the selenoenzymes from Sh14 and the wild-type enzyme, as they are able not only to catalyze the reduction of CuOOH but also displayed considerable catalytic activity when assayed with the H_2_O_2_/GSH substrate system. Noteworthy, the magnitude of this catalytic activity is higher than that exhibited by a natural GPX (Table 2) [19,42].

**Figure 8:**
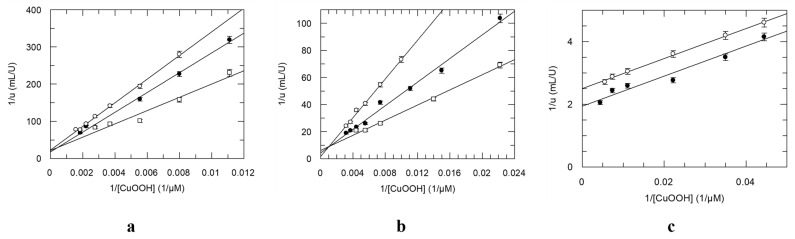
(**a**) Double-reciprocal plots for the reduction of CuOOH by GSH [0.05 mM (○), 0.1 mM (●) και 1.0 mM (□)] catalyzed by *Gm*GSTU4-4. (**b**) Double-reciprocal plots for the reduction of CuOOH by GSH [0.05 mM (○), 0.2 mM (●) και 1.5 mM (□)] catalyzed by Sh14. (**c**) Double-reciprocal plots for the reduction of CuOOH by GSH [0.5 mM (○) and 1 mM (●)] catalyzed by SeSh14. Data points are expressed as means ± SD (n > 3).

Previous investigation has established that the kinetic mechanism of the wild-type *Gm*GSTU4-4 using the CDNB and GSH as substrates obeys a rapid equilibrium random sequential bi–bi model, in agreement with the mechanism displayed by other native GSTs [43,44,45]. The kinetic mechanism of GSTs is isoenzyme- and substrate-dependent. To assess whether the *Gm*GSTU4-4, Sh14, and SeSh14 enzymes follow the same rapid equilibrium random sequential bi–bi kinetic mechanism, using CuOOH as a substrate, initial velocity studies were undertaken (Figure 8). The results suggested that when CuOOH was used as a variable substrate with several fixed concentrations of GSH, an intersecting pattern of Lineweaver–Burk plots were obtained for the *Gm*GSTU4-4 and Sh14 enzymes (Figure 8a,b). On the contrary, kinetics analysis of the SeSh14, using H_2_O_2_ as a substrate, showed parallel Lineweaver–Burk plots (Figure 8c), a characteristic of the Ping–Pong kinetic mechanism, similar to that operated by the natural GPX [42,46,47]. This finding indicates that SeSh14 has evolved to an enzyme with kinetics and an activity profile similar to the natural GPX.

## 4. Conclusions

The GST scaffold is a highly flexible platform for designing novel catalytic activities, especially in the context of detoxifying xenobiotic compounds. In the present work, the combination of directed evolution with chemical modification allowed for the creation of a semisynthetic enzyme with high GPX activity. The GPX activity of the evolved enzyme was increased by several folds after the conversion of the active-site Ser to Sec with chemical modification. Noteworthy, the same Ping–Pong mechanism as the natural GPX was observed when the kinetic behavior of the SeSh14 was investigated. The results of the present study shed light on the common GPX and GST evolution history, supporting the notion that both enzymes have arisen from a single thioredoxin-like progenitor.

## Figures and Tables

**Table 1:** Kinetic parameters of the wild-type *Gm*GSTU4-4, the Sh14 variant, and the SeSh14 enzyme for GSH, CuOOH, and H_2_O_2_. The wild-type *Gm*GSTU4-4 and the Sh14 variant did not display activity towards H_2_O_2_.

Enzyme	k_cat_ (min^−1^) (CuOOH)	k_cat_ (min^−1^) (H_2_O_2_)	Κ_m_ (μΜ) GSH	Κ_m_ (μΜ) CuOOH	Κ_m_ (μΜ) H_2_O_2_	k_cat_/Κ_m_ (×10^−3^) (μΜ^−1^·min^−1^) (CuOOH)	k_cat_/Κ_m_ (×10^−3^) (μΜ^−1^·min^−1^) (H_2_O_2_)
*Gm*GSTU4-4	10.7 ± 1.0	-	3.6 ± 0.8	454.3 ± 83.6	-	23.6	-
Sh14	35.6 ± 3.7	-	1.2 ± 0.1	255.8 ± 46.4	-	139.2	-
SeSh14	1898.7 ± 79.9	5659.4 ± 442.3	999.3 ± 232.1	29.3 ± 5.3	83.8 ± 14.7	64,802	67,510

**Table 2:** Specific activity of the *Gm*GSTU4-4, Sh14, Ser13Ala mutant, and the selenoenzymes Se*Gm*GSTU4-4, SeSh14, and SeSer13Ala using three substrate systems: CDNB/GSH, CuOOH/GSH, and H_2_O_2_/GSH. Enzyme assays were performed in triplicate and in all cases, the standard deviation was <5%.

Enzyme	Substrate System	Specific Activity (μmol·min^−1^·mg^−1^)
*Gm*GSTU4-4	CDNB/GSH	11.2
	CuOOH/GSH	0.5
	H_2_O_2_/GSH	ND ^1^
Se*Gm*GSTU4-4	CDNB/GSH	ND
	CuOOH/GSH	90.0
	H_2_O_2_/GSH	67.5
Sh14	CDNB/GSH	48.4
	CuOOH/GSH	2.9
	H_2_O_2_/GSH	ND
SeSh14	CDNB/GSH	ND
	CuOOH/GSH	349.2
	H_2_O_2_/GSH	244.5
Ser13Ala	CDNB/GSH	0.04
	CuOOH/GSH	ND
	H_2_O_2_/GSH	ND
SeSer13Ala	CDNB/GSH	ND
	CuOOH/GSH	0.3
	H_2_O_2_/GSH	0.7
SerGST T2-2 (seleno rat GSTT2-2)	CuOOH	23.00 ^2^
	H_2_O_2_	102.00 ^2^
GPx (human erythrocytes)	H_2_O_2_	100.00 ^3^

^1^ NA: No activity was detected. ^2^: Reported in [8]. ^3^: Reported in [19].

## Data Availability

Data are contained within the article or Supplementary Material.

## Supplementary Materials

The following supporting information can be downloaded at: https://www.mdpi.com/article/10.3390/antiox13010041/s1, Table S1: the sequence of primers used in mutagenesis reactions at amino acid position 114.
