# Supplementary material for: Repurposing Glutathione Transferases: Directed Evolution Combined with Chemical Modification for the Creation of a Semisynthetic Enzyme with High Hydroperoxidase Activity
Source: Antioxidants (Basel). 2023 Dec 25;13(1):41. doi: 10.3390/antiox13010041 (PMC10812501; doi:10.3390/antiox13010041)
Supplement: Supplementary file 1 [file antioxidants-13-00041-s001.zip › antioxidants-2762962-supplementary.pdf]

Table S1. The sequence of primers used in mutagenesis reactions at amino acid position 114.

**CysSh14Trp**	5'- GGAAGATT**TGG**ACATCAAAAGC-3'
**CysSh14Trp**	5'- CCTTTTGATGT**CCA**AATCTTCC-3'
**CysSh14Phe**	5'- GGAAGATT**TTT**ACATCAAAAGC-3'
**CysSh14Phe**	5'- CCTTTTGATGT**AAA**AATCTTCC-3'
**CysSh14Leu**	5'- GGAAGATT**TTA**ACATCAAAAGC-3'
**CysSh14Leu**	5'- CCTTTTGATGT**TAA**AATCTTCC-3'
**CysSh14Ala**	5'- GGAAGATT**GCT**ACATCAAAAGC-3'
**CysSh14Ala**	5'- CCTTTTGATGT**AGC**AATCTTCC-3'
